# Combining Gender, Work, and Family Identities: The Cross-Over and Spill-Over of Gender Norms into Young Adults’ Work and Family Aspirations

**DOI:** 10.3389/fpsyg.2016.01781

**Published:** 2016-11-17

**Authors:** Loes Meeussen, Jenny Veldman, Colette Van Laar

**Affiliations:** Center for Social and Cultural Psychology, Department of Psychology, University of LeuvenLeuven, Belgium

**Keywords:** identity compatibility, identity management, gender, work, family, social norms

## Abstract

The current study investigates how descriptive and prescriptive gender norms that communicate work and family identities to be (in)compatible with gender identities limit or enhance young men and women’s family and career aspirations. Results show that young adults (*N* = 445) perceived gender norms to assign greater compatibility between female and family identities and male and work identities than vice versa, and that young men and women mirror their aspirations to this traditional division of tasks. Spill-over effects of norms across life domains and cross-over effects of norms across gender-groups indicated that young women, more than young men, aimed to ‘have it all’: mirroring their career ambitions to a male career model, while keeping their family aspirations high. Moreover, young women opposed traditional role divisions in the family domain by decreasing their family aspirations in face of norms of lower family involvement or higher career involvement of men. Conversely, in line with traditional gender roles, young men showed lower family aspirations in the face of strong male career norms; and showed increases in their career aspirations when perceiving women to take up more family roles. Young men’s family aspirations were, however, more influenced by new norms prescribing men to invest more in their family, suggesting opportunities for change. Together, these findings show that through social norms, young adults’ gender identity affects aspirations for how to manage the co-presence of their work and family identities. Altering these norms may provide leverage for change to allow both men and women to combine their multiple identities in an enriching way.

## Introduction

Many people on a daily basis manage the combination of two identities central in their lives: their work identity and their family identity. The co-presence of these identities may be experienced as conflictual or as enriching, with important consequences for well-being, health, and performance (Allen et al., 2000; Van Steenbergen and Ellemers, 2009). We argue that people’s gender identity impacts the way men and women cope with the co-presence of work and family identities differently. More specifically, decisions about engaging in or refraining from work and family roles are influenced by prevailing gender norms in one’s social context – norms that indicate what roles are and should be for men and women, and as such, communicate to what extent male and female identities are (in)compatible with work and family identities (Eagly et al., 2000; Eagly and Karau, 2002). Advancing research that shows norms are highly influential within a gender group and life domain (Major, 1994; Wood and Eagly, 2009; Brown and Diekman, 2010), we argue that due to the intertwined nature of gender, work, and family identities, norms may ‘spill-over’ from one life domain to the other (e.g., family norms affecting career aspirations) and ‘cross-over’ from one gender-group to the other (e.g., norms for men affecting women’s aspirations).

Laying bare such inter-identity dynamics of norms allows us to identify potential barriers to and leverages for change toward greater compatibility between male and family identities, and between female and work identities, with various benefits. Increasing male opportunities in the family domain is beneficial for men’s psychological and physical health for their children’s upbringing, and for reducing women’s second shift (for a review, see Croft et al., 2015). Increasing female opportunities in the work domain is beneficial for women’s feelings of life-enrichment and well-being (Van Steenbergen et al., 2007; Jacob, 2008), for performance of organizations (Carter et al., 2003; Herring, 2009), and to decrease children’s gender stereotypes (Davis and Greenstein, 2009).

### The Importance of Gender Norms: Communicating (in) Compatibility with Family and Work Identities

Gender norms indicate which roles are for men and which roles are for women (Eccles, 1994; Eagly et al., 2000; Eagly and Karau, 2002). With regard to work and family identities, normative beliefs persist that male identities are more compatible with work identities, and thus that men should be -or are- better at being the provider; and that female identities are more compatible with family identities, and thus that women should be -or are- better at being the caregiver (Riggs, 1997; Etaugh and Folger, 1998; Eagly et al., 2000; Hodges and Park, 2013). Research has shown that such gender norms affect aspirations, decisions, and behavior as people internalize social norms (Major, 1994; Wood and Eagly, 2009) and experience social and personal benefits when they adhere to gender norms and punishments when they do not (Brown and Diekman, 2010; Diekman et al., 2011). Replicating such research, we expect that people’s family and career aspirations will be influenced by norms for their own gender with regard to that same life domain. Extending this research, we argue that work, family, and gender identities are so intertwined that gender norms are not only influential within gender-groups and life domains, but also from one life domain to the other (referred to as spill-over effects, cf. Bakker and Demerouti, 2013) and from one gender-group to the other (referred to as cross-over effects).

#### Gender Norms Spill-Over from One Life Domain to the Other

Many people manage both work and family identities on a daily basis, sometimes experiencing a conflicting and sometimes an enriching combination (Carlson et al., 2000; Hakanen et al., 2011). Given this close link between work and family identities, we argue that norms about the compatibility between one’s gender and one life domain will also affect one’s aspirations in the other life domain, indicating how people combine work and family identities. We predict that particularly for women, norms regarding one domain will reinforce aspirations in the other domain, as research indicates that women more than men experience the combination of a career and family identity as enriching and rewarding (Van Steenbergen et al., 2007). Moreover, societal changes lead women especially to increasingly consider “having it all,” that is, pursuing a successful career and family life (Hoffnung, 2004). Indeed, in the last decades, women’s career involvement has increased greatly (England, 2010, 2011), while at the same time women continue to have the main responsibility for family tasks (Hochschild and Machung, 2012; European Commission, 2014).

#### Gender Norms Cross-Over from One Gender Group to the Other

In addition to spillover from one domain to another, we predict there also to be cross-overs from one gender group to another. Partners need to find a division of work and family tasks between them, since the less one partner does, the more the other will have to fill in (Belsky and Kelly, 1994; Biehle and Mickelson, 2012). As such, within heterosexual relationships male and female identities are very much intertwined (Elder, 1998). Given this close link, we argue that norms about the compatibility between one gender-group and work or family will also affect the other gender group’s aspirations.

First, gender norms for the other gender may cross-over to influence people’s aspirations *within one life domain*, indicating that people may mirror themselves to roles for the other gender. For example, women may increase their career aspirations the more they perceive men to invest in their career. As gender roles are increasingly egalitarian (Botkin et al., 2000), the other gender becomes a more relevant comparison group for one’s own aspirations. We predict that such relations will especially occur for women, since their roles have changed more toward those of men than vice versa (Eagly and Diekman, 2003; Croft et al., 2015). Moreover, matching their aspirations to male norms means an opportunity for self-improvement for women (Corcoran et al., 2011) since roles typically occupied by men tend to be higher in status and authority than roles typically occupied by women (Eagly et al., 2000; England, 2010, 2011).

Second, gender norms for the other gender in one domain may cross-over to influence one’s aspirations *in the other domain*, indicating how work and family roles are negotiated between partners. For example, men may increase their career aspirations the more they perceive women to invest in their family (i.e., influence in traditional direction) or women may decrease their family aspirations the more they perceive men to invest in their careers (i.e., influence in non-traditional direction). We predict that women more than men will oppose traditional gender divisions, because such divisions are most disadvantageous for them in a society in which women’s career involvement has greatly increased while men’s involvement in family tasks has remained behind, thus saddling women with a second shift (Bianchi et al., 2000; Laflamme et al., 2002; Pleck and Masciadrelli, 2004; Bartley et al., 2005; Hochschild and Machung, 2012). Conversely, men are likely to oppose traditional divisions less, or even follow them, since they experience less disadvantages of such divisions and may thus be more likely to follow system-justifying beliefs that what “is” is what “ought” to be (Heider, 1958; Sidanius and Pratto, 1999; O’Brien and Major, 2009).

To examine these relationships, we had young adults anticipate their life as working parents and respond to how they imagined their opportunities and responsibilities in the family and work domains. Young adulthood (18–30 years old; Rindfuss, 1991) is a crucial life stage in which decisions are made regarding ways of managing the combination of work and family identities (Brown and Diekman, 2010; Weisgram et al., 2010), and a stage in which instigating possible change toward gender equality in the future is most likely. We examined both descriptive and prescriptive norms. Descriptive norms indicate what men and women currently do (Eagly, 1987; Heilman, 2001, 2012; Prentice and Carranza, 2002). They reflect what young adults see in their social context: to what extent do men and women currently take up family and career roles? We included descriptive norms as people’s behavior is shaped by what they encounter in their social surrounding (Oishi and Graham, 2010), and young adults’ choices regarding families and careers are influenced by what they have seen around them (Croft et al., 2014; Gerson, 2010). Prescriptive norms meanwhile indicate what men and women should do (Eagly, 1987; Heilman, 2001, 2012; Prentice and Carranza, 2002). They reflect what is deemed desirable: to what extent men and women should invest in work and family roles. Both descriptive and prescriptive norms are important, since what people should do according to their social environment may differ from what people actually do. Also, what people see around them and what they believe to be appropriate may both push social change, and this change could push toward more traditional or more egalitarian gender roles.

## Materials and Methods

### Participants

Participants were young adults at the University of Leuven in Belgium who participated in an online study about ‘their present and future life.’ Belgium can be seen as an average Western country in terms of gender equality: Its gender equality index is comparable to other Western European countries such as France or Germany, and in-between Eastern European countries scoring in the lower range and Scandinavian countries scoring in the higher range of gender equality (European Institute for Gender Equality, 2015). Compared to the United States, Belgium scores somewhat higher on gender equality with a 10th place worldwide based on the global gender gap index (Unites States 20th place; World Economic Forum, 2014) and a 9th place worldwide based on the gender inequality index (United Stated 47th place; United Nations Development Programme, 2014).

Participants took part in this study in exchange for course credits or a chance to win a number of €9–35 prizes in a lottery. Participants were recruited through student mailing lists, social media, flyers in university buildings, and promotion talks during lectures at various faculties. Of the 522 students who completed the survey instrument, a number were excluded as they did not fit the population of interest: seven were excluded from the sample because they were over 30 years old and hence no longer fit the group of young adults (Rindfuss, 1991); 37 were excluded as they indicated not being heterosexual (which meant gender norms would not be expected to cross-over for them in the same way), and 33 were excluded because they indicated that they did not want to have children (which made the family aspiration measures about childcare not applicable to them). Of the resulting sample of 445 participants, 37.8% were male and 62.2% female with a mean age of 19.3 (*SD* = 1.92). The sample reflected the typical university campus: Participants came from a wide variety of majors, such as law, economics and business, engineering, medicine, humanities, and social sciences. Most participants self-identified as Belgian (93.4%), and 19.1% (also) identified with another group (mostly other European countries).

### Measures

All items were answered on a 7-point Likert scale from (1) *strongly disagree* to (7) *strongly agree*. Measures were scored such that higher scores indicate stronger scores on the concept.

#### Own Family and Career Aspirations

To assess own future family and career aspirations, participants were asked to imagine their life in 15 years. Given that participant’s mean age was 19 years, this meant that they indicated how they saw their family and career investment around the age of 34, an age at which many people in the Western world are combining a career with a household and taking care of one or more young children (Mathews and Hamilton, 2009). Participants’ *own family aspirations* were measured with four items (two childcare items, two household items): ‘15 years from now… I will make time to take care of all the needs of my children’; ‘I will not really concern myself with my children’s upbringing’ (reversed); ‘I will spend considerable time making sure that the household is in order’; and ‘I will spend little time on household tasks’ (reversed). *Own career aspirations* were measured with two items ‘15 years from now, I will spend time on building a successful career’ and ‘15 years from now, I will spend little time on my career’ (reversed).

#### Descriptive Norms Regarding Family and Career Investment

Descriptive norms about men’s family investment, men’s career investment, women’s family investment, and women’s career investment were measured with the same items as for own aspirations but rephrased to inquire about what the average man or woman in Belgium does (cf. Gill, 2004). For instance, an item measuring descriptive norms regarding men’s family investment was ‘The average man in Belgium spends little time on household chores’ (reversed) and an item measuring descriptive norms regarding women’s career investment was ‘The average woman in Belgium spends time on building a successful career.’ It was emphasized that we were interested in what participants believed the average man or woman in Belgium did, not what they thought about this personally.

#### Prescriptive Norms Regarding Family and Career Investment

Similarly, the same items were adapted to measure prescriptive family and career norms for men and for women by asking what they *should do* (cf. Heilman, 2001, 2012). Every item was measured twice, asking both what men prescribe (e.g., ‘The average man in Belgium believes that men should make time to take care of all the needs of their children’) and what women prescribe (e.g., ‘The average woman in Belgium believes that men should make time to take care of all the needs of their children’). The norms for women (prescribed by women and men) were then combined into one scale, as were the norms for men (prescribed by women and men). In this way, we ensured that the items measured consensual norms rather than participants’ own opinion as to what men and women should do.

**Table 1** provides reliability scores, means, standard deviations, and correlations between all measures. Items were worded rather strongly to ensure it would not be too easy to fully agree or disagree, thus avoiding ceiling or floor effects. The means on the scales (ranging between 3.88 and 5.67 on 7-point scales) indicated this was successful. As described above, for each measurement two items were used (one of which was always a reversed item): two items that measured household investment, two items that measured childcare investment, and two items that measured career investment. As exploratory factor analyses for all of the measures (i.e., own aspirations, descriptive and prescriptive norms for men and women) revealed a two-factor solution with career investment on the one hand and childcare and household investment on the other (57.15–63.89% explained variance), childcare and household investment were combined into one family investment scale.

**Table 1:** Reliability scores, means, standard deviations, and correlations between all measures for male and female participants.

Variable	Reliability	*M*(*SD*) Male participants	*M*(*SD*) Female participants	1	2	3	4	5	6	7	8	9	10
(1) Own family aspirations	α = 0.65	5.09 (0.84)	5.35 (0.83)		0.27^∗∗∗^	0.25^∗∗∗^	0.29^∗∗∗^	0.47^∗∗∗^	0.24^∗∗∗^	0.23^∗∗∗^	0.27^∗∗∗^	0.38^∗∗∗^	0.15^∗^
(2) Own career aspirations	*r* = 0.45^∗∗∗^	5.37 (1.03)	5.37 (0.89)	-0.05		0.06	0.32^∗∗∗^	0.19^∗∗^	0.32^∗∗∗^	0.08	0.38^∗∗∗^	0.19^∗∗^	0.22^∗∗∗^
(3) Descriptive family norm for men	α = 0.67	4.02 (0.81)	3.91 (0.87)	0.08	0.05		0.00	0.13^∗^	0.33^∗∗∗^	0.44^∗∗∗^	0.09	0.03	0.25^∗∗∗^
(4) Descriptive career norm for men	*r* = 0.44^∗∗∗^	5.06 (0.89)	5.53 (0.85)	-0.03	0.17^∗^	-0.14		0.38^∗∗∗^	0.27^∗∗∗^	0.19^∗∗∗^	0.50^∗∗∗^	0.38^∗∗∗^	0.28^∗∗∗^
(5) Descriptive family norm for women	α = 0.77	5.55 (0.81)	5.68 (0.82)	0.24^∗∗^	0.27^∗∗∗^	0.25^∗∗^	0.27^∗∗∗^		-0.02	0.26^∗∗∗^	0.48^∗∗∗^	0.66^∗∗∗^	0.04
(6) Descriptive career norm for women	*r* = 0.45^∗∗∗^	4.33 (0.89)	4.49 (0.96)	0.16^∗^	-0.01	0.09	0.22^∗∗^	0.04		0.31^∗∗∗^	0.27^∗∗∗^	0.05	0.53^∗∗∗^
(7) Prescriptive family norm for men	α = 0.69	4.65 (0.71)	4.62 (0.70)	0.30^∗∗∗^	0.15	0.35^∗∗∗^	0.18^∗^	0.36^∗∗∗^	0.16		0.22^∗∗∗^	0.27^∗∗∗^	0.54^∗∗∗^
(8) Prescriptive career norm for men	α = 0.71	5.18 (0.81)	5.52 (0.80)	0.10	0.35^∗∗∗^	0.05	0.47^∗∗∗^	0.43^∗∗∗^	0.19^∗^	0.26^∗∗^		0.61^∗∗∗^	0.37^∗∗∗^
(9) Prescriptive family norm for women	α = 0.86	5.21 (0.88)	5.63 (0.82)	0.28^∗∗^	0.16	0.15	0.30^∗∗∗^	0.51^∗∗∗^	0.21^∗∗^	0.37^∗∗∗^	0.61^∗∗∗^		0.09
(10) Prescriptive career norm for women	α = 0.66	4.53 (0.80)	4.51 (0.83)	0.04	0.07	0.14	0.17^∗^	0.09	0.33^∗∗∗^	0.37^∗∗∗^	0.36^∗∗∗^	0.18^∗^	


*Correlations above left diagonal are for female participants, correlations below the diagonal for male participants. ^∗∗∗^*p* < 0.001, ^∗∗^*p* < 0.01, ^∗^*p* < 0.05.*

## Results and Discussion

### Traditional and Changing Gender Norms

Before looking into the relation between gender norms and young adults’ own aspirations, we examined how young males and females perceived descriptive and prescriptive gender norms regarding family and career identities. Supporting the notion that consensual norms rather than personal opinions or preferences were being assessed, within-group reliability indices were all above the 0.70 criterion (James et al., 1984; LeBreton and Senter, 2008) indicated that young adults (*M*_Rwg_ = 0.82, *SD* = 0.03), as well as young males (*M*_Rwg_ = 0.79, *SD* = 0.02) and young females separately (*M*_Rwg_ = 0.83, *SD* = 0.03), showed considerable consensus on what the descriptive and prescriptive norms are for what men and women do and should do in terms of family and work. **Table 2** presents differences and similarities between young males’ and females’ in their perceptions of the norms (independent samples *t*-tests: top half of table), as well as differences between descriptive and prescriptive norms (paired samples *t*-tests: bottom half of table).

**Table 2:** Difference tests investigating how descriptive and prescriptive norms about career and domestic investment differ from one another for men and women (bottom half of table), and in the perception of male and female participants (final column top half of table).

Norm	Male participants *M*(*SD*)	Female participants *M*(*SD*)	Independent samples *t*-tests: Difference male vs. female participants
Descriptive family norm for men	4.02 (0.81)	3.91 (0.87)	1.25
Descriptive career norm for men	5.06 (0.89)	5.53 (0.85)	-5.25^∗∗∗^
Descriptive family norm for women	5.55 (0.81)	5.68 (0.82)	-1.57
Descriptive career norm for women	4.33 (0.89)	4.49 (0.96)	-1.67^†^
Prescriptive family norm for men	4.65 (0.71)	4.62 (0.70)	0.40
Prescriptive career norm for men	5.18 (0.81)	5.52 (0.80)	-4.09^∗∗∗^
Prescriptive family norm for women	5.21 (0.88)	5.63 (0.82)	-4.88^∗∗∗^
Prescriptive career norm for women	4.53 (0.80)	4.51 (0.83)	0.23

**Paired samples *t*-tests: Difference between norms**			

(1) Diff descriptive family vs. career norm for men	-9.95^∗∗∗^	-21.92^∗∗∗^	
(2) Diff descriptive family vs. career norms for women	12.53^∗∗∗^	15.38^∗∗∗^	
(3) Diff descriptive family norms for men vs. women	-18.78^∗∗∗^	-26.18^∗∗∗^	
(4) Diff descriptive career norms for men vs. women	8.04^∗∗∗^	15.56^∗∗∗^	
(5) Diff prescriptive family vs. career norms for men	-7.00^∗∗∗^	-15.70^∗∗∗^	
(6) Diff prescriptive family vs. career norms for women	7.68^∗∗∗^	16.54^∗∗∗^	
(7) Diff prescriptive family norms for men vs. women	-7.62^∗∗∗^	-18.12^∗∗∗^	
(8) Diff prescriptive career norms for men vs. women	8.63^∗∗∗^	18.06^∗∗∗^	
(9) Diff descriptive vs. prescriptive family norm for men	-8.83^∗∗∗^	-13.91^∗∗∗^	
(10). Diff descriptive vs. prescriptive career norm for men	-1.62	0.22	
(11). Diff descriptive vs. prescriptive family norm for women	4.82^∗∗∗^	1.13	
(12) Diff descriptive vs. prescriptive career norm for women	-2.51^∗^	-0.45	


**^∗∗∗^p* < 0.001, *^∗∗^p* < 0.01, *^∗^p* < 0.05, ^†^*p* < 0.10.*

Specifically, the results showed that generally, young males and females had a relatively traditional consensus about patterns in descriptive and prescriptive norms. Both young males and females believed that norms show greater compatibility between male and work identities and between female and family identities: As indicated in the lower half of **Table 2**, they believed norms to indicate that men currently do (difference #1) and should (difference #5) invest more in their career than in their family, and that women currently do (difference #2) and should (difference #6) invest more in their family than in their career. Similarly, the task-division between partners was perceived to be rather traditional, as young adults believed norms to indicate that men currently do (difference #4) and should (difference #8) invest more in their career than women, and that women currently do (difference #3) and should (difference #7) invest more in their family than men. Moreover, differences between young males and females’ norm perceptions (top half **Table 2**, last column) indicated that young females estimated men’s current career investment significantly higher than young males do, and they perceived prescriptive norms to indicate more strongly that men should invest in a career and that women should invest in a family. Thus, young females perceived gender norms to be even more traditional than young males, which could be explained by the fact that women experience more disadvantages from traditional gender divisions than men (Eagly et al., 2000; England, 2010, 2011; Gerson, 2010).

Importantly, there was also some indication of change toward greater gender equality when comparing descriptive with prescriptive norms: Young males and females believed that norms indicate that men should invest more in family tasks than they currently do (bottom half of **Table 2**, difference #9), and young men believed that norms indicate women should invest less in family tasks (difference #11) and more in their career (difference #12) than they currently do. Thus, both young males and females appear ready for some movement toward less traditional role divisions; which could be fostered so that this is translated into actual behavior when these young adults become parents.

### Gender Norms and Young Adults’ Own Family and Career Aspirations

We then examined the relationship between gender norms and young adults’ aspirations. For this, we conducted hierarchical regressions for young males and young females separately, predicting their family or work aspirations (while controlling for work and family aspirations, respectively) from (1) norms for their own gender within the same life domain; (2) norms for their own gender in the other life domain (i.e., spill-over); and (3) norms for the other gender (i.e., cross-over). **Tables 3** and **4** present the results of the final regression models for career and family aspirations, respectively, as well as R square difference tests for each step. While gender norms were related to one another (as would be expected), VIF indices between 1.20 and 2.45 and Tolerance indices between 0.41 and 0.83 indicated no multicollinearity concerns (cf., Dormann et al., 2013).

**Table 3:** Hierarchical regression models predicting young male and female participants’ career aspirations from different gender norms (standardized coefficients).

	Young males				Young females			
		
Predictor of career aspirations	Model 1	Model 2	Model 3	Model 4	Model 1	Model 2	Model 3	Model 4
Own family aspirations	-0.07	-0.11	-0.14^†^	-0.14	0.26^∗∗∗^	0.19^∗∗^	0.12^†^	0.14^∗^
**Norms within gender and domain**								
Descriptive career norm for own gender		-0.01	-0.01	-0.03		0.25^∗∗∗^	0.27^∗∗∗^	0.23^∗∗^
Prescriptive career norm for own gender		0.36^∗∗∗^	0.34^∗∗∗^	0.41^∗∗∗^		0.06	0.05	0.002
**Spill-over from family to career**								
Descriptive family norm for own gender			0.01	-0.01			0.10	0.04
Prescriptive family norm for own gender			0.10	0.12			0.07	-0.09
**Cross-over from other gender**								
Descriptive career norm for other gender				-0.02				0.11^†^
Prescriptive career norm for other gender				-0.09				0.28^∗∗∗^
Descriptive family norm for other gender				0.20^∗^				-0.05
Prescriptive family norm for other gender				-0.19^†^				-0.07
*R^2^*	0.01	0.13	0.14	0.19	0.07	0.14	0.16	0.24
*R*^2^ difference		0.13^∗∗∗^	0.01	0.05		0.08^∗∗∗^	0.02^†^	0.08^∗∗∗^


*^∗∗∗^*p* < 0.001, ^∗∗^*p* < 0.01, ^∗^*p* < 0.05, ^†^*p* < 0.10.*

**Table 4:** Hierarchical regression models predicting young male and female participants’ family aspirations from different gender norms (standardized coefficients).

	Young males				Young females			
		
Predictor of family aspirations	Model 1	Model 2	Model 3	Model 4	Model 1	Model 2	Model 3	Model 4
Own career aspirations	-0.07	-0.12	-0.14^†^	-0.14	0.26^∗∗∗^	0.17^∗∗^	0.10^†^	0.12^∗^
**Norms within gender and domain**						
Descriptive family norm for own gender		-0.02	-0.05	-0.10		0.37^∗∗∗^	0.39^∗∗∗^	0.37^∗∗∗^
Prescriptive family norm for own gender		0.32^∗∗∗^	0.32^∗∗∗^	0.27^∗∗^		0.11	0.09	0.19^∗^
**Spill-over from career to family**						
Descriptive career norm for own gender			-0.14	-0.19^∗^			0.21^∗∗^	0.16^∗^
Prescriptive career norm for own gender			0.13	0.01			-0.01	0.04
**Cross-over from other gender**						
Descriptive family norm for other gender				0.18^†^				0.17^∗∗^
Prescriptive family norm for other gender				0.16				-0.05
Descriptive career norm for other gender				0.16^†^				0.08
Prescriptive career norm for other gender				-0.11				-0.17^∗^
*R^2^*	0.01	0.10	0.12	0.20^∗∗∗^	0.07	0.26	0.30	0.33^∗∗∗^
*R^2^* difference		0.10^∗∗^	0.02	0.08^∗^		0.19^∗∗∗^	0.04^∗∗^	0.03^∗^


*^∗∗∗^*p* < 0.001, ^∗∗^*p* < 0.01, ^∗^*p* < 0.05, ^†^*p* < 0.10.*

#### Norms for Own Gender within Life Domains: Young Adults Mirror Their Aspirations to (Traditional and Changing) Gender Norms

Looking at the role of norms for one’s own gender within life domains, the results showed that young adults’ career aspirations were related to career norms for their own gender: As **Table 3** shows, the more young males believed that men should invest in their career, the more they aspired to invest in their career themselves (β = 0.41 *p* < 0.001). The more young females perceived women to currently invest in their career, the more they aspired to invest in their career themselves (β = 0.23, *p* = 0.001). Similarly, as shown in **Table 4**, young adults’ family aspirations were related to family norms for their gender: The more young females perceived women to currently invest and the more they perceived norms to indicate they should invest in their family, the more they aspired to invest in their family themselves (β = 0.37, *p* < 0.001 and β = 0.19, *p* = 0.019, respectively). Also, the more young males believed that men should invest in their family, the more they aspired to invest in their family themselves (β = 0.27, *p* = 0.004).

Thus, young adults mirrored their own aspirations to rather traditional gender norms, indicating that gender equality in both the family and career domain may not occur rapidly unless change is fed. The findings point to opportunities for change especially in the family domain: young men’s own family aspirations were related to prescriptive rather than descriptive norms. Given that both young males and females perceived norms to ascribe that men should invest more in their family than what they currently invest, gender equality in the family domain is likely to be improved when such changing gender norms are made salient. Also, the advantages of men taking part in family tasks for men’s psychological and physical health, for women’s well-being, for relationship satisfaction, and for children’s adjustment (Biehle and Mickelson, 2012; Croft et al., 2014, 2015) could be highlighted and communicated more. More generally, since young adults mirror their own aspirations to the norms which they perceive for their own gender-group, investing in altering their perceptions of gender norms to be less traditional is likely to feed social change. For instance, such change may be initiated by more non-traditional gender role models in education and the mass media (Hogben and Waterman, 1997; Greenwood and Lippman, 2009) and through children’s home environment (Goldberg et al., 2012; Croft et al., 2014).

#### Spill-Over Effects of Norms between Life Domains: Young Women But Not Young Men Want it All?

Next, we examined spill-over effects of own-gender norms on young adults’ aspirations from one life domain to the other. Results showed that family aspirations were positively related to own-gender career norms for women, while they were negatively related for men: That is, the more young women perceived women to currently invest in a career, the more they aspired to also invest in family tasks (β = 0.16, *p* = 0.015). Conversely, the more young men perceived men to currently invest in a career, the less they aspired to invest in family tasks (β = -0.19, *p* = 0.040). This gender difference was also visible in the relations between family aspirations and career aspiration, which were significant and positive for women and non-significant and negative for men.

These findings suggest that young women, more than or even in contrast to young men, ‘want it all’ (cf. Hoffnung, 2004): they want to pursue high aspirations in the family as well as in the career domain. This corresponds to the societal trend that while their participation in the labor market and ‘agentic’ type tasks has increased substantially, women continue to have the main responsibility for family and communal tasks (Diekman et al., 2011; Hochschild and Machung, 2012; European Commission, 2014). On the one hand, wanting it all may be beneficial for women, given that they experience the combination of work and family roles as even more enriching than do men (Van Steenbergen et al., 2007). On the other hand, these high aspirations may also make women more vulnerable to work-family conflict once they enter parenthood (Nomaguchi and Milkie, 2003; Hodges and Park, 2013).

While young women’s family aspirations were positively related to female career norms, their career aspirations were not related to female family norms. This difference may be a result of high expectations for mothers (Thurer, 1995; Hays, 2003; Johnston and Swanson, 2006): a strong focus on women’s careers may bring about feelings of guilt and hence push aspirations for their mother identity, while a stronger focus on family does not bring about the same guilt for their work identity and thus does not push career identity (Rotkirch and Janhunen, 2010; Liss et al., 2013).

Young males’ family aspirations were negatively related to male career norms, but their career aspirations were not related to male family norms. This suggests that when experiencing conflict between family and career roles, young males would follow more traditional gender role patterns, decreasing family aspirations for career aspirations rather than the other way around. This is consistent with research showing that men are penalized for decreasing career time for family reasons (Rudman and Mescher, 2013).

#### Cross-Over Effects of Other Gender Norms within Life Domains: Young Women’s Aspirations Follow Male Norms

We next examined cross-over effects of norms by investigating relationships between young males’ and females’ aspirations and norms for the *other* gender. With regard to cross-over effects *within* one life domain, as expected young females’ but not young males’ aspirations were related to gender norms for the other gender. Specifically, the results show that young females’ family aspirations were higher the more they believed men currently invested in family tasks (β = 0.17, *p* = 0.006). This may suggest that young females aim for a ‘tit for tat’ strategy toward a more equal partner division of family tasks in a world where men spend only half the time on family tasks women do (Bianchi et al., 2000; Bartley et al., 2005). Moreover, young females related their career aspirations to a more male career model: The more they believed norms to ascribe men should invest in a career, the higher their own career aspirations (β = 0.28, *p* = 0.001)^1^. This also fits with the notion that male career roles tend to be higher in status than female roles, and therefore, matching their aspirations to male career norms also means an increasing opportunity for self-improvement for women (Eagly et al., 2000; England, 2010, 2011).

#### Cross-Over Effects of Other Gender Norms between Life Domains: Women More Than Men Oppose Traditional Task-Divisions

With regard to cross-over effects *between* life domains, opposite patterns emerged for young males and females: The more young females believed norms to ascribe men should invest in a career, the less they themselves aspired to invest in family tasks (β = -0.17, *p* = 0.034) – and the more they also aspired a career, as described above. This suggests women may increasingly actively oppose traditional role divisions when males have a strong emphasis on careers. Conversely, the more young males believed women invest in family tasks, the more they aspired to invest in their career (β = 0.20, *p* = 0.046), reinforcing a traditional role division with men as providers and women as caregivers, in line with system-justifying beliefs that what “is” is what “ought” to be (Heider, 1958; Sidanius and Pratto, 1999; O’Brien and Major, 2009). Thus, relating these patterns to the descriptive results, young females perceived norms to be more traditional and they opposed traditional role divisions more than young males^2^. These gender differences correspond to the fact that women experience more disadvantages from traditional gender divisions than men (Eagly et al., 2000; England, 2010, 2011; Gerson, 2010); and they provide opportunities for change: Fostering young women’s resistance to traditional gender divisions by decreasing family aspirations may decrease a second shift and maternal gatekeeping behaviors for women (Allen and Hawkins, 1999; Hochschild and Machung, 2012); and our results indicate that this, in turn, could also change men’s focus on career over family roles.

More generally, the results showed that young females’ aspirations more than young males’ were related to prevailing gender norms (i.e., more significant relationships and higher R squares among females- thus more variance in their aspirations explained by gender norms). This is in accordance with previous research showing that especially young females assume that the choices they make need to fit their social context (Oyserman and James, 2011). This difference between young males and females was also due to the fact that women’s aspirations were influenced more by male gender norms than vice versa, which, as noted, may reflect an opportunity for self-improvement for women (Eagly et al., 2000; England, 2010, 2011).

### Limitations and Directions for Future Research

The current study investigated how descriptive and prescriptive gender norms that communicate work and family identities to be (in)compatible with gender identities limit or enhance young men and women’s family and career aspirations. Advancing research that shows norms are highly influential within a gender group and life domain (Major, 1994; Wood and Eagly, 2009; Brown and Diekman, 2010), we argued that due to the intertwined nature of gender, work, and family identities, norms may ‘spill-over’ from one life domain to the other (e.g., family norms affecting career aspirations) and ‘cross-over’ from one gender-group to the other (e.g., norms for men affecting women’s aspirations). Together, the findings show that through social norms, young adults’ gender identity affects aspirations for how to manage the co-presence of their work and family identities. Altering these norms may provide leverage for change to allow both men and women to combine their multiple identities in an enriching way.

One potential limitation of the current study is that we cannot automatically generalize data collected in a particular norm setting to other nations or norm settings. However, as indicated earlier, Belgium is comparable to other Western countries in terms of gender inequality (United Nations Development Programme, 2014; World Economic Forum, 2014; European Institute for Gender Equality, 2015). Also, while the levels of young adults’ aspirations and gender norms may differ between countries (Crompton and Lyonette, 2006; Fahlén, 2014), the relations between norms and aspirations – the main focus of this paper – are likely to be generalized more broadly.

In this paper, we studied young adults’ aspirations for their future work and family life. While we believe young adults to be an important sample because decisions regarding work and family life are shaped before people actually become working parents, we do not wish to imply that these aspirations will necessarily directly translate into the future lives of these individuals. Young adults’ hopes, choices, and decisions are not fixed but continue to change, shaped by personal attitudes and factors in their social environment (cf. Hoffnung and Williams, 2013), an important one of which, as we show, is gender norms. Also, many of these young adults may not yet have a very clear notion of how they will balance their family and career responsibilities and how they will negotiate tasks with their partner. Therefore, we did not ask participants directly about the challenges in combining or dividing tasks, but more implicitly about their aspirations and the norms in their environment to then infer the combinations of life domains and partners from the spill-over and cross-over analyses. The current sample were young adults attending university, a more highly educated segment of the general population. As is typically the case for higher educated groups, this sample is likely to represent where gender role change or maintenance is headed. Our results indicated that these young adults still showed attachment to traditional gender roles, as well as clear elements of change.

A limitation of the current study is its correlational design, which does not allow for firm causal conclusions from the results. While it is possible that young adults’ family and career aspirations are *projected onto* rather than *influenced* by their perception of gender norms, this alternative explanation is less likely for a number of reasons: First, the finding that young males and females showed strong consensus in their perceptions of gender norms but show very different relations between these norms and their own aspirations speaks against the idea that young adults would base their perception of gender norms on their own aspirations. Similarly, the spill-over and cross-over effects of norms are hard to explain through reverse causation. For instance, if young females project their career aspirations onto gender norms, then it is unlikely that they would also project their aspirations onto career norms for men instead of only on career norms for women. Moreover, the fact that gender norms affect people’s own aspirations and behavior has been well documented in previous research (Major, 1994; Brown and Diekman, 2010; Diekman et al., 2011). Still, future studies could further investigate the causal link between gender norms and young adults’ aspirations using longitudinal or experimental designs. For instance, it would be fruitful to investigate whether the relationships that suggest leverage for change toward gender equality can be manipulated: Do men increase their family aspirations when they are informed that changing norms ascribe men to do so? Do they become more interested in family roles when the status of such roles is highlighted? Do women decrease their family aspirations when norms ascribing a strong career focus for men are highlighted? Moreover, one could examine actual partner negotiations with regard to family and work responsibilities: How do partners come to a particular division of tasks and to what extent are gender norms implicitly or explicitly at play during such negotiations? We are examining these questions in ongoing research.

## Conclusion

In sum, this paper examines how young men and women expect to fill in their future multiple identities, and how this relates to the norms they perceive in the environment around them. The results showed that young men and women’s management of the co-occurrence of their work and family identities is differentially influenced by norms for their and the other gender identity. While norms were perceived to be rather traditional, results also revealed several areas in which there is leverage for change toward gender equality in both the family and work domains, such that men and women can regulate their gender, professional, and family identities as an enriching rather than conflicting combination.

## Ethics Statement

The study has been approved by Social and Societal Ethics Committee, University of Leuven, Belgium. Before starting the questionnaire, participants agreed to an informed consent that provided the general research aims. They were informed that participation was voluntary and could be stopped at any moment during the study; and that their responses are anonymous and treated confidentially. Moreover, they were provided with room for questions and comments as well as all contact information of the researchers and the ethical committee – and they received a full debriefing at the end of the study.

## Author Contributions

LM, JV, and CvL contributed to the development of hypotheses, data collection, interpretation of results, and this manuscript. LM conducted the statistical analyses.

## Conflict of Interest Statement

The authors declare that the research was conducted in the absence of any commercial or financial relationships that could be construed as a potential conflict of interest.

## Footnotes

1 The marginally significant relation between descriptive career norms for men and young female’s career aspirations showed a similar trend: the more women perceived men to currently invest in a career, the higher their own career aspirations (β = 0.11, *p* = 0.091).

2 Three effects did suggest a trend away from traditionalism for young males, though not significant at the 0.05 level: Young males’ family aspirations were higher the more they perceived women to currently invest in a family (β = 0.18, *p* = 0.071; suggesting a tendency ‘to do their part’) and the more they perceived women to currently invest in a career (β = 0.16, *p* = 0.057; suggesting they would want to enable a future partner’s career). Moreover, the more young males perceived norms to indicate women should invest in a family, the lower their own career aspirations (β = -0.19, *p* = 0.085; suggesting a trend to oppose traditional divisions, similar to our findings for young females).
